# Targeted physical therapy for gouty arthritis: a first-principles frequency-screening study

**DOI:** 10.3389/fphys.2026.1868909

**Published:** 2026-10-06

**Authors:** Yuqi Xia, Yifan Guan, Peilin Li, Yitong Wang, Yawen Li, Mingyan Xie, Zhe Wang, Kai Li, Peng Zhang

**Affiliations:** 1 Shandong Provincial Key Laboratory of Nuclear Science, Nuclear Energy Technology and Comprehensive Utilization, Weihai Frontier Innovation Institute of Nuclear Technology, Shandong University, Weihai, China; 2 School of Space Science and Technology, Shandong University, Weihai, China; 3 SDU-ANU Joint Science College, Shandong University, Weihai, China

**Keywords:** density functional theory simulation, gouty arthritis treatment, mid-infrared laser therapy, monosodium urate monohydrate, photon–phonon resonant absorption

## Abstract

**Introduction:**

Gouty arthritis is caused by the deposition of monosodium urate monohydrate (MSUM) crystals in joints. Current drug therapies are often limited by side effects, and invasive procedures carry associated risks. This study aims to provide a theoretical basis for local physical treatment of gout by identifying specific mid-infrared frequencies for irradiating MSUM crystals via photon-phonon resonant absorption.

**Methods:**

The vibrational spectra of crystalline MSUM were simulated using first-principles density functional theory. The infrared and Raman peaks were mapped to their corresponding vibrational modes, and relative infrared activities were analyzed. The infrared spectrum of an anhydrous monosodium urate model (anhydrous MSU) was also calculated.

**Results:**

Analysis of the vibrational spectra revealed infrared characteristic peaks at 1637 and 1696 cm^-^¹ for MSUM and at 1627 and 1705 cm^-^¹ for anhydrous MSU. This finding provides the theoretical foundation for scanning 1627–1705 cm^-^¹ (48.78–51.11 THz) to resonantly excite the crystals.

**Discussion:**

The irradiation of MSUM crystals with a mid-infrared laser near 50 THz is proposed for initial frequency-scanning experiments. A optical fiber placed near deeper deposits could shorten the distance traveled through tissue. However, experimental validation is required to determine suitable irradiation conditions and assess safety and efficacy.

## 1 Introduction

Gouty arthritis is an inflammatory disorder caused by disrupted uric acid metabolism and the resulting abnormal deposition of monosodium urate monohydrate (MSUM) crystals in synovial joints and surrounding tissues (Kuo et al., 2015; Zhou et al., 2022). The hallmark pathological features of this disease include localized acute inflammation, which is commonly observed in big toes, ankles, and knees. Gout is not confined to joint pathology, and its pathogenesis is closely linked to systemic metabolic dysregulation. Clinical studies indicate that gout patients exhibit markedly higher risks of developing metabolic syndrome (Puig and Martínez, 2008; Kuo et al., 2016), cardiovascular diseases (Abbott et al., 1988; Krishnan et al., 2006), and renal disorders (Yu et al., 2012). Etiologically, the pathogenesis of gout is multifactorial, involving hyperuricemia, genetic predisposition (Blumberg, 1965), dietary factors (Choi et al., 2004), medications, comorbidities, and lead exposure.

Pharmacotherapy is the primary treatment for gouty arthritis and can be categorized as acute-attack or long-term urate-lowering therapy. Medications for acute gout attacks include nonsteroidal anti-inflammatory drugs (NSAIDs), colchicine, and corticosteroids (Shrestha et al., 1995; Janssens et al., 2008; Gaffo and Saag, 2009; Richette and Bardin, 2010; Taylor, 2017; Mirkov, 2018). All NSAIDs can exert adverse effects. For example, indomethacin and phenylbutazone (Smyth and Percy, 1973), which are frequently used as first-line treatments for acute gout, cause gastrointestinal toxicity, nephrotoxicity, or gastrointestinal bleeding (Cronstein and Terkeltaub, 2006). Colchicine can be administered orally. Oral administration can causes diarrhea (Emmerson, 1996; Bonnel et al., 2002; Terkeltaub et al., 2010). Systemic corticosteroids are a short-term alternative when NSAIDs or colchicine are ineffective or contraindicated (Janssens et al., 2008).

Long-term urate-lowering therapy relies on uricosuric agents and xanthine oxidase inhibitors. Commonly used uricosurics include probenecid, sulfinpyrazone, and benzbromarone, which, however, may promote urate crystal deposition in renal tubules and urinary tracts and thus cause renal colic or dysfunction (Emmerson, 1996). Additionally, benzbromarone can cause hepatotoxicity and is therefore banned in numerous countries (Terkeltaub, 2017). Allopurinol, the clinically used xanthine oxidase inhibitor, primarily poses hypersensitivity and cutaneous reaction risks (Singer and Wallace, 1986; Chao and Terkeltaub, 2009; Ramasamy et al., 2013). The intermittent intake or discontinuation of medications lowering serum urate concentrations can lead to acute gout and gouty stone recurrence; therefore, urate-lowering therapy must be lifelong.

Given the contraindications and side effects of pharmacotherapy, noninvasive and highly selective physical therapies are highly sought after. Laser therapy is effective for treating arthritis caused by urate deposition (Soriano et al., 2006; Rodriguez-Silva et al., 2020; Bartoli et al., 2021) but poses problems related to optimization of parameters (e.g., wavelength, dose, power density, exposure duration, and treatment site). As MSUM crystal deposition is the direct cause of gouty arthritis (Chih et al., 2016; Li et al., 2020), this work investigates the vibrational spectra of crystalline MSUM. Based on the photon–phonon resonant absorption (PPRA) mechanism, a physical process in which photons transfer energy to specific phonon modes of the crystal lattice to induce resonance, we identify candidate laser frequencies for initial irradiation experiments on MSUM crystals.

## 2 Simulation methods

The CASTEP first-principles density functional theory (DFT) code (Clark et al., 2005) within the Materials Studio software was used to optimize the crystal structure of MSUM and simulate its vibrational spectrum. The best match with the experimental results was obtained using the RPBE (revised Perdew-Burke-Ernzerhof) functional of the generalized gradient approximation (Kalkura et al., 1995; Li et al., 2020) as implemented with the Tkatchenko-Scheffler (TS) dispersion correction. The energy and self-consistent-field convergence tolerances were set to $1\;\times \;10^{-9}$ eV/atom. A 1 × 2 × 4 k-point mesh, cutoff energy of 830 eV, and norm-conserving pseudopotential with a linear response approximation were used. The peaks in the simulated infrared (IR) and Raman spectra were assigned to various vibrational modes.

MSUM belongs to the triclinic crystal system ( a = 10.888 Å,b = 9.534 Å, c = 3.567 Å, α = 95.06°, β = 99.47°, γ =97.17°) (Mandel and Mandel, 1976; Boone-Kukoyi et al., 2019). The urate anions are hydrogen-bonded to each other and intimately aligned along the edges of their purine rings, forming two-dimensional sheets of purine rings parallel to the plane. Adjacent sheets coalesce via π–π stacking interactions to establish a three-dimensional lattice. Sodium cations occupy interlaminar regions and attractively interact with aromatic π-electrons. Structural water molecules form hydrogen bonds with deprotonated nitrogen sites while coordinating sodium cations through oxygen-mediated ionic interactions. For comparison, a monosodium urate model without structural water (hereafter, anhydrous MSU) was optimized and its vibrational spectrum was calculated. We compared the spectra of the two optimized structures to see whether the anhydrous model also absorbs within the range selected for MSUM. This comparison would help select frequencies for testing whether absorption continues after structural water loss.

## 3 Results

The 38-atom MSUM unit cell contained two MSUM molecules (NaC_5_H_3_N_4_O_3_·H_2_O), which yielded 111 ( 38 × 3 − 3) vibrational normal modes. **Figure 1** presents the simulated IR and Raman spectra of MSUM. The centrosymmetric structure of MSUM prohibits simultaneous changes in dipole moment and polarizability during vibrations, resulting in complementary IR and Raman activities. Using dynamic process analysis, we categorized all modes as skeletal deformations or water rotations, bending vibrations, and stretching vibrations.

**Figure 1:**
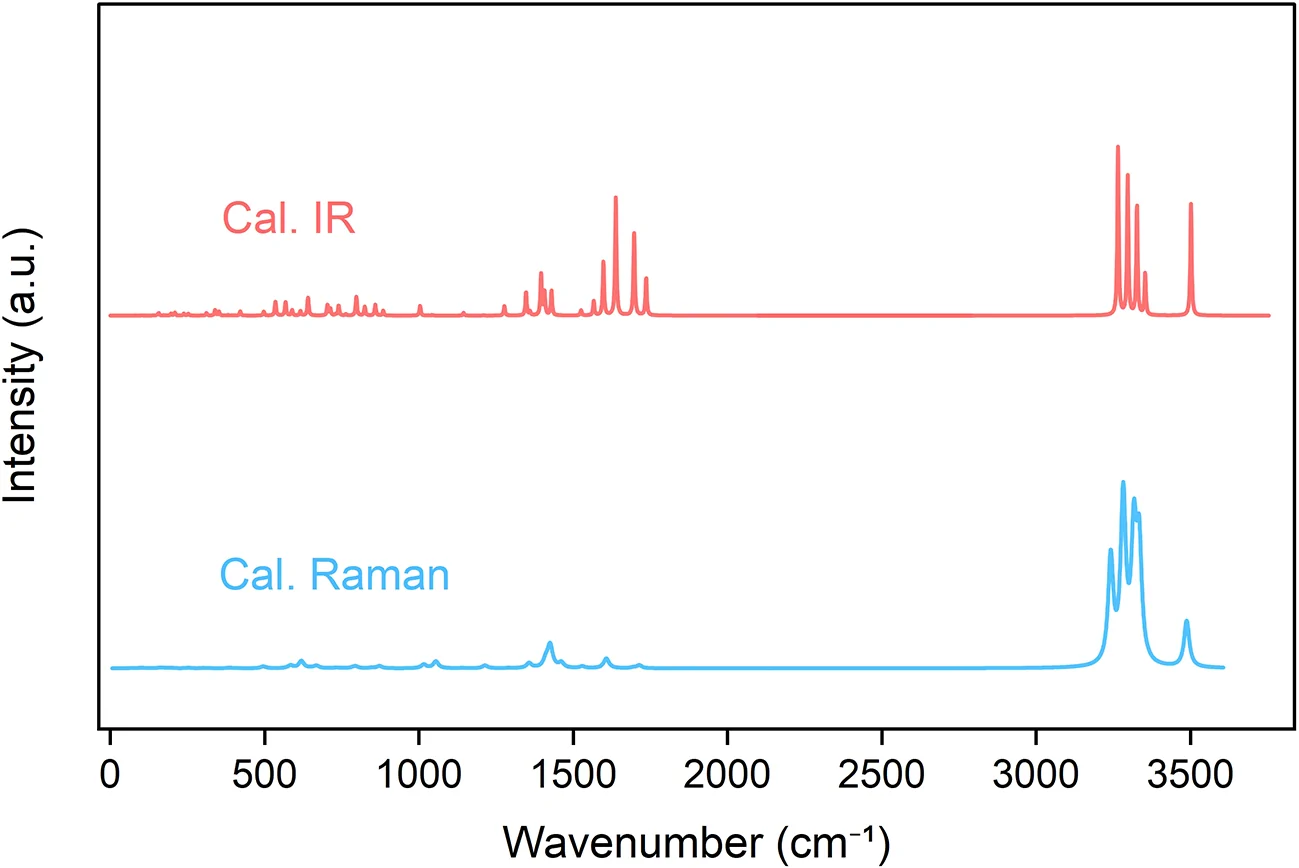
Simulated infrared (IR; red) and Raman (blue) spectra of monosodium urate monohydrate (MSUM). Although the two spectra are similar, the corresponding vibrational modes are complementary.

Peaks below 588 cm^-^¹ were categorized as water rotations, sodium-ion motions, and skeletal deformations. **Figure 2** illustrates the atomic vibration patterns corresponding to four high-intensity peaks in this region, namely the IR peaks at 209 and 534 cm^-^¹ and Raman peaks at 493 and 584 cm^-^¹. However, the intensities of these peaks are very low compared with those of higher-wavenumber peaks. The peak at 209 cm^-^¹ corresponds to sodium-ion translation, while that at 493 cm^-^¹ corresponds to a skeletal deformation, and those at 534 and 584 cm^-^¹ represent water rotations. For a given vibrational mode, the center of mass of the primitive cell remains static, which means that minute motions balancing the main motion can be observed. Given that the motion amplitude of an atom is inversely proportional to its mass, the motion of oxygen atoms is difficult to recognize.

**Figure 2:**
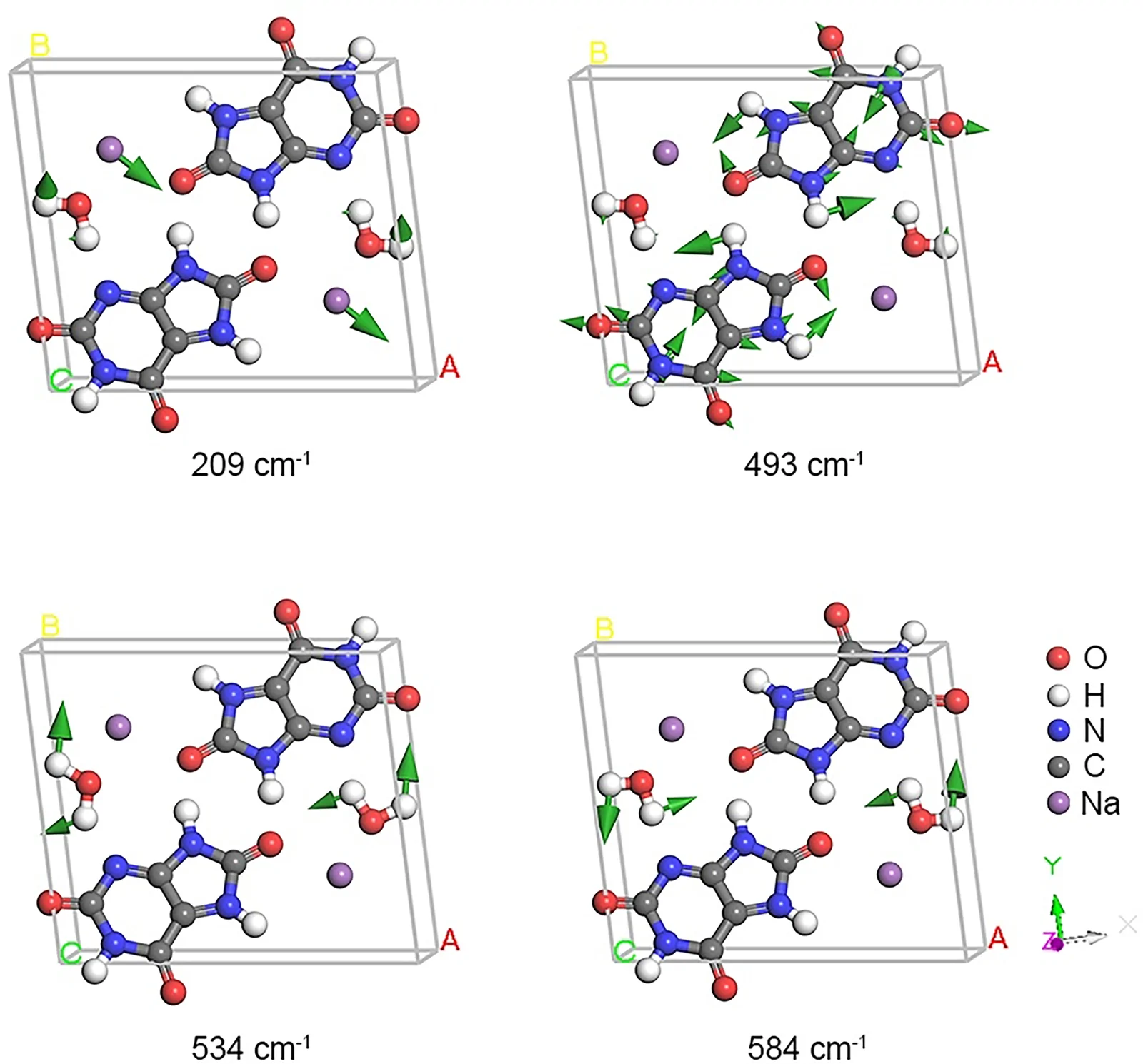
Atomic vibration patterns corresponding to four vibrational modes of MSUM in the low-energy region of <588 cm^-^¹. Green arrows represent motion directions, and arrow dimensions represent motion amplitudes.

Peaks in the range of 615–1735 cm^-^¹ were assigned to bending vibrations. **Figure 3** shows the atomic vibration patterns corresponding to four high-intensity IR peaks at 796, 1597, 1637, and 1696 cm^-^¹. The experimental IR spectrum of MSUM features peaks at 787 cm^-^¹ (N–H bending of the purine ring) and 1647 cm^-^¹ (water bending) (Li et al., 2020), which can be mapped to the simulated IR peaks at 796 and 1637 cm^-^¹, respectively. The simulated peaks at 1597 and 1696 cm^-^¹ were primarily assigned to the bending of N–H groups. These two peaks superimpose to form the strongest absorption band in this area. The experimental Raman spectrum of MSUM features three high-intensity peaks at 1011, 1062, and 1208 cm^-^¹ (Li et al., 2020), which can be matched to the simulated Raman peaks at 1015, 1054, and 1213 cm^-^¹, respectively, exhibiting good agreement with the experimental results.

**Figure 3:**
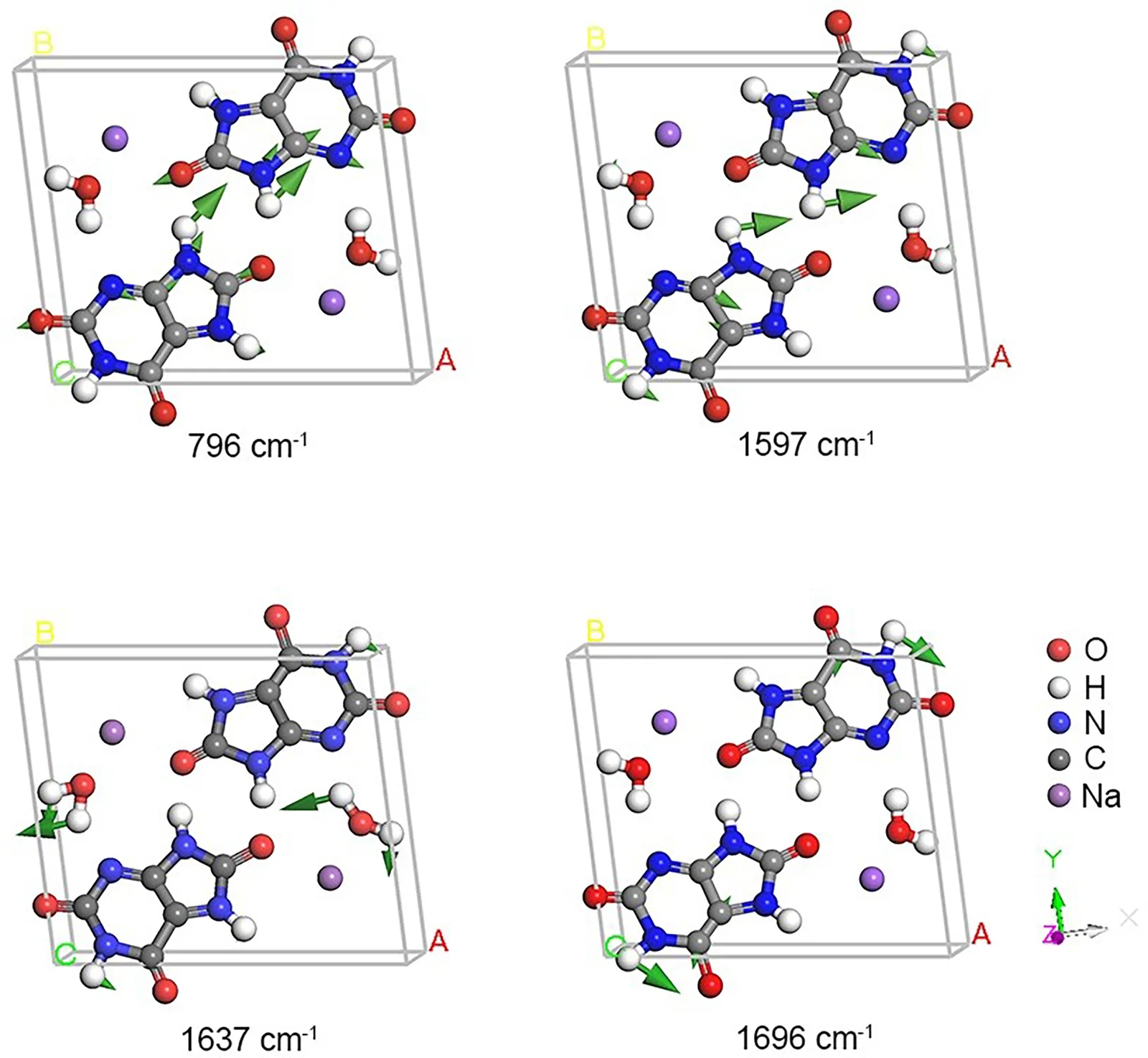
Atomic vibration patterns corresponding to four bending modes of MSUM. Green arrows represent motion directions, and arrow dimensions represent motion amplitudes. The peaks at 796, 1597 and 1696 cm^-^¹ are assigned to N–H bending vibrations in the purine ring. The peak at 1637 cm^-^¹ is water bending mode.

Peaks above 3241 cm^-^¹ were ascribed to stretching modes (**Figure 4**). The peaks at 3325 cm^-^¹ (IR) and 3333 cm^-^¹ (Raman) were identified as the O–H stretches of water. The dominant IR peak at 3295 cm^-^¹ was identified as an asymmetric N–H stretch, with its symmetric counterpart appearing at 3281 cm^-^¹ in the Raman spectrum and representing the strongest Raman peak. The Raman peak at 3487 cm^-^¹ (**Figure 1**) was assigned to O–H stretching, with its IR counterpart observed at 3500 cm^-^¹. It is worth noting that the calculated relative intensities differ from some experimental reports—specifically, the stretching vibrational modes (~3300 cm^-^¹) appear more intense in the simulation than in practice. This discrepancy is attributed to environmental factors (hydrogen bonding, hydration effects) and measurement conditions (sample morphology) that are not fully captured in the ideal periodic DFT model. DFT calculations are highly reliable for predicting vibrational frequencies, even though intensities may show greater variability. We have compared our calculated frequencies with reported experimental data (Li et al., 2020) to verify the assignments. We consider the bending region near 1666 cm^-^¹ as a candidate for initial PPRA scans.

**Figure 4:**
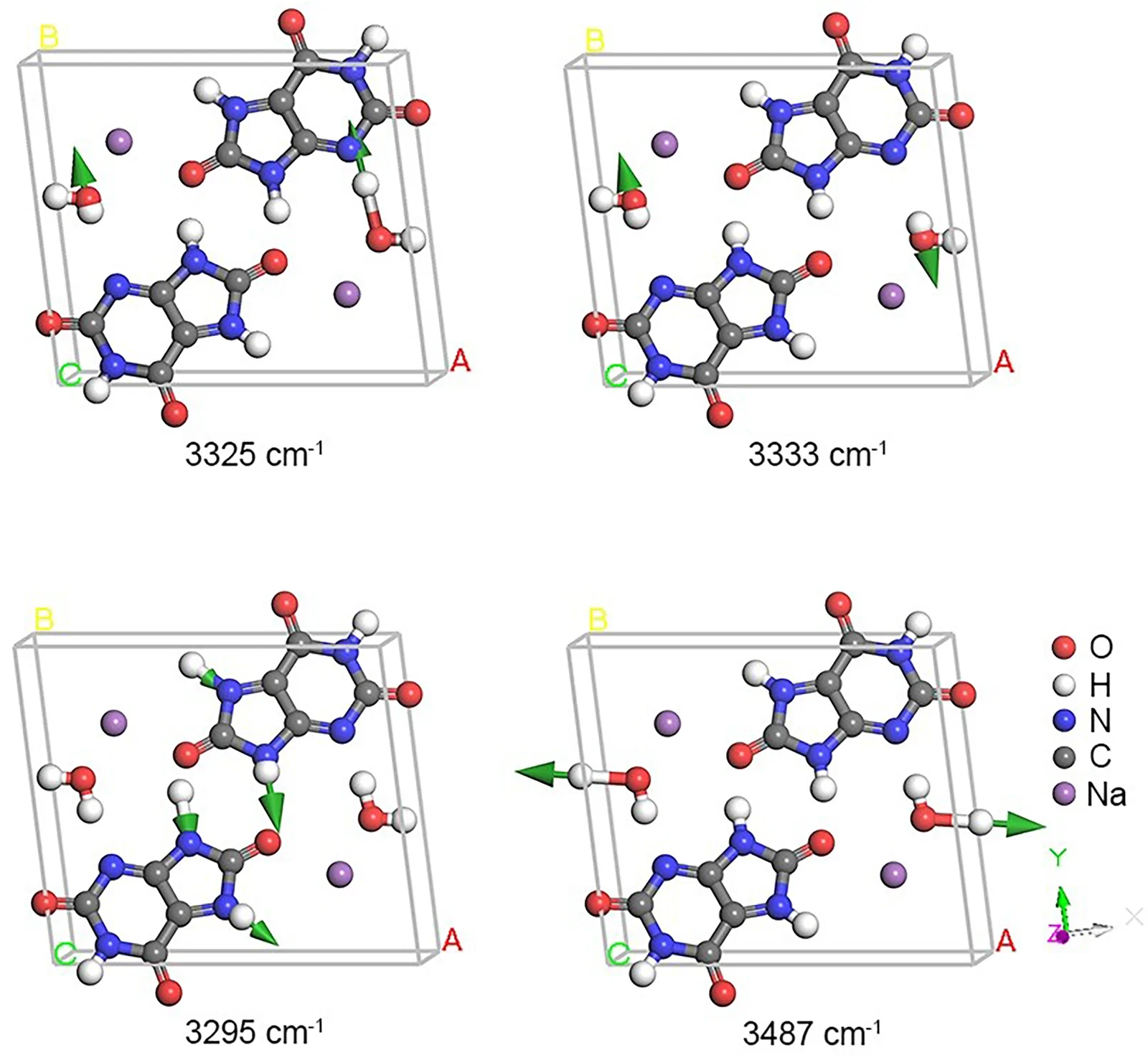
Atomic vibration patterns corresponding to four stretching vibrations of MSUM. Green arrows represent motion directions, and arrow dimensions represent motion amplitudes. The peaks at 3325, 3333, and 3487 cm^-^¹ correspond to different water stretching combinations, and the peak at 3295 cm^-^¹ corresponds to N–H stretching vibrations.

Additionally, we simulated the vibrational spectra of the optimized MSU crystal and assigned the key peaks through the dynamic analysis of vibrational modes. As shown in **Figure 5**, the most prominent absorption band does not shift during the dehydration of MSUM to MSU. **Figure 6** shows the atomic vibration patterns corresponding to four close vibrational modes, with the peaks at 1637 and 1696 cm^-^¹ representing the water-bending and N–H-bending modes of MSUM, respectively, and those at 1627 and 1705 cm^-^¹ corresponding to the N–H bending modes of MSU. Consequently, the frequency corresponding to 1666 cm^-^¹ (49.95 THz, photon energy 206.56 meV) was selected as the initial center for a PPRA scan covering 1627–1705 cm^-^¹. This range corresponds to 48.78–51.11 THz, 5.87–6.15 μm, and photon energies of 201.72–211.39 meV, with a total frequency span of approximately 2.34 THz. The irradiation of MSUM by a tunable laser covering this range could excite these IR-active modes. As the optimized anhydrous MSU model still strongly absorbs in this band, anhydrous material with a comparable structure could continue to absorb energy after structural water loss.

**Figure 5:**
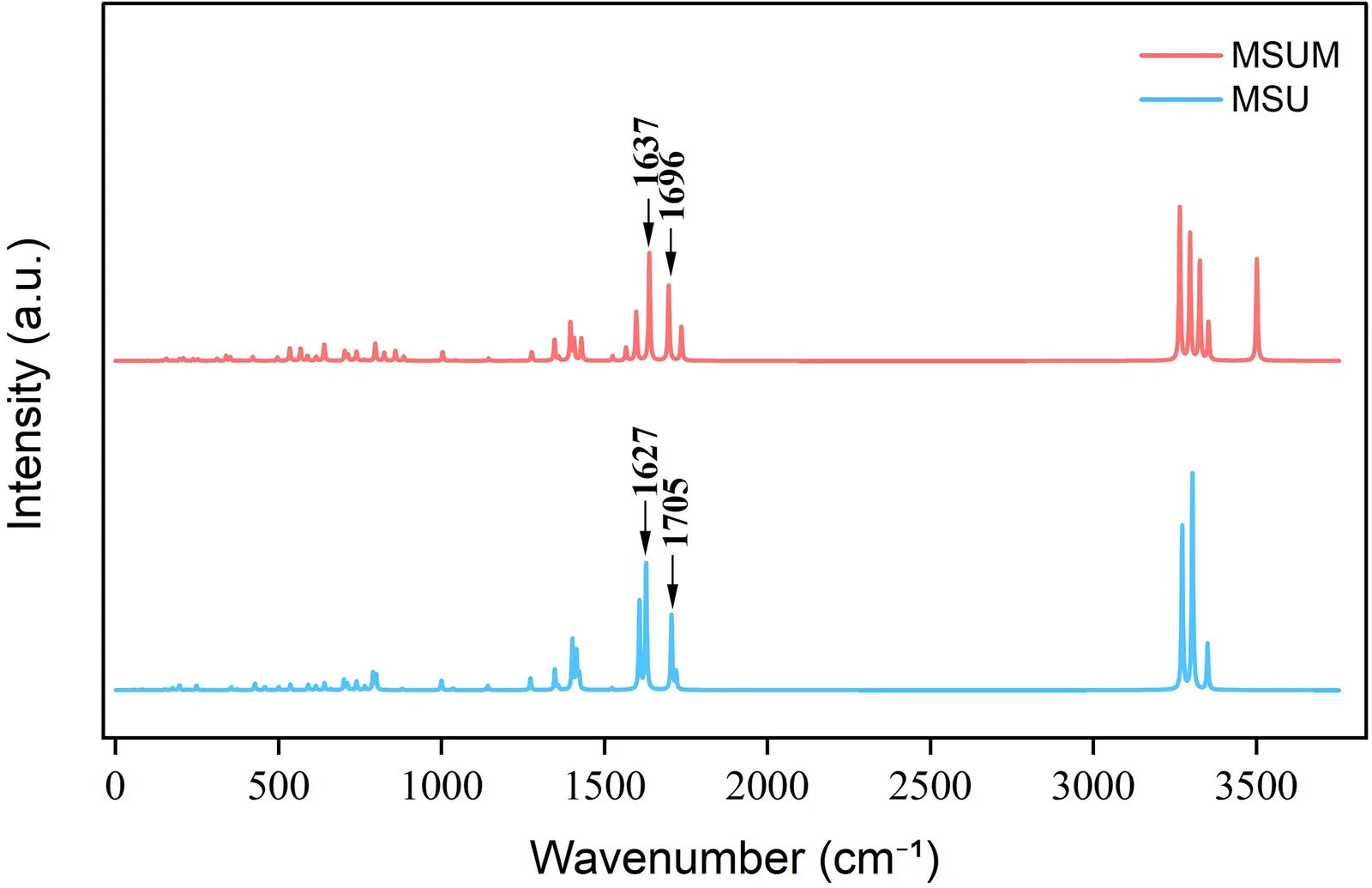
Simulated IR spectra of MSUM (red) and monosodium urate (MSU; blue). The peaks at 1637 and 1696 cm^-^¹ in the spectrum of MSUM change to the peaks at 1627 and 1705 cm^-^¹ in the spectrum of MSU. This wavenumber range corresponds to a frequency of ~50 THz.

**Figure 6:**
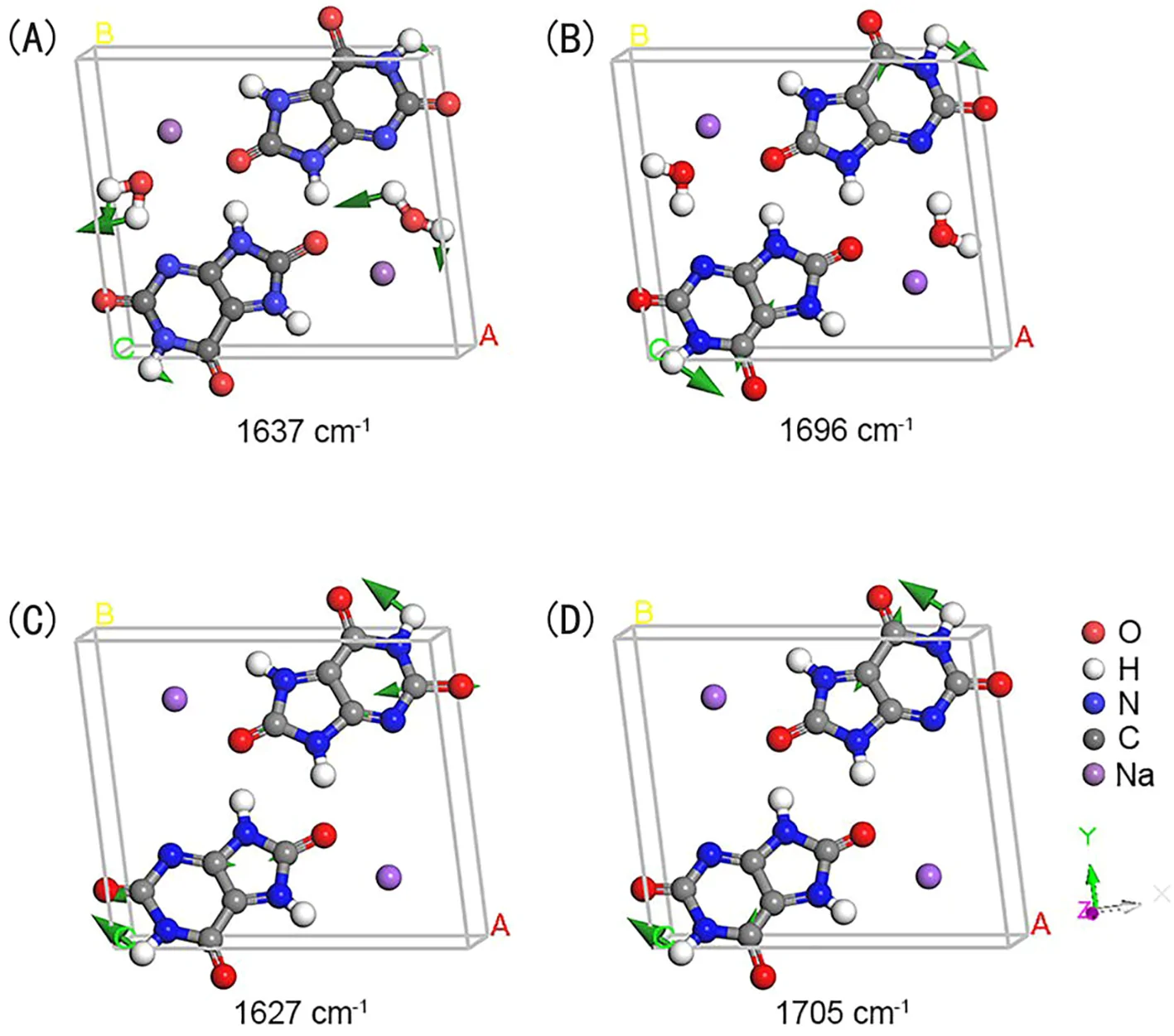
Atomic vibration patterns corresponding to four close peaks. Green arrows represent motion directions, and arrow dimensions represent motion amplitudes. **(A)** Water bending vibrations (1637 cm^-^¹) and **(B)** N–H bending vibrations (1696 cm^-^¹) of MSUM. **(C, D)** N–H bending vibrations of MSU.

## 4 Discussion

Continued irradiation after structural water loss could promote lattice disorder or fragmentation and alter the dissolution behavior of the remaining solid. A reduction in the amount of deposited crystals would depend on their dissolution in the surrounding fluid. Dissolution varies with the liquid environment and the properties of the crystals (Tamasi et al., 2013). Crystals prepared by different methods can also cause different inflammatory responses (Yan et al., 2023), so changes in the properties of the irradiated crystals may also affect inflammation. In the planned experiments, measurements of crystal phase, particle size and shape, and dissolved urate would show how much solid remains after irradiation. Subsequent biological tests would examine the effects of the treated material on local inflammation.

For deposits below the tissue surface, water absorption in the 5.87–6.15 μm region limits the distance that light can travel through water-rich tissue (Hale and Querry, 1973). In laser lithotripsy, optical fibers have been used to deliver light directly to stones inside the body (Buxbaum et al., 2018). Positioning the fiber tip close to the crystals would shorten this distance and concentrate irradiation near the deposit. Studies of hollow-glass waveguides have demonstrated transmission near the selected band (Shen et al., 2001), and infrared-transmitting fibers have delivered mid-infrared radiation in liquid environments (Mackanos et al., 2003). In a joint, some light would be lost as it travels through the fiber and the liquid between the tip and the crystals, reducing the energy that reaches the deposit. The design would also depend on fiber flexibility, whether the device can be sterilized, and how the tip is protected from liquid entering it.

Future tissue experiments would monitor crystal changes and nearby tissue temperature with the fiber tip placed close to the deposit. Conventional Ho:YAG lithotripsy relies mainly on photothermal decomposition (Chan et al., 1999). Compared with nearby off-resonance irradiation, tuning the laser to an IR-active crystal mode might produce a similar crystal change with less incident energy and less heating of the surrounding tissue. Absorption by the surrounding medium would also contribute to the temperature rise. The link between light absorption and stone removal has been examined in lithotripsy experiments (Shalini et al., 2020). The planned tests would compare the candidate frequencies with nearby off-resonance frequencies while keeping the fiber position and spot size the same and using comparable pulse settings and irradiation times. Recording crystal changes, the energy reaching the sample, and temperature would show whether frequency selection can reduce the energy needed for a similar change in the crystals and limit heating. Temperature and irradiation time together describe the degree and duration of tissue heating (Sapareto and Dewey, 1984). These measurements could guide adjustments to laser settings, irradiation time, fluid cooling, and fiber position in tissue studies (Wollin et al., 2018).

The calculations describe ideal periodic crystals within harmonic and linear-response approximations. Temperature, anharmonicity, vibrational relaxation, crystal surfaces and defects, and differences in particle size and shape may shift or broaden the bands. The surrounding fluid and tissue would also affect how much light reaches the crystals and how the absorbed energy is distributed.

The planned experimental work would begin by measuring the infrared spectra of synthetic MSUM, anhydrous MSU after confirmation of its crystal phase, and the surrounding liquid. These spectra would guide scans within 1627–1705 cm^-^¹ and at nearby off-resonance frequencies, with the range adjusted to the measured bands. Water content, crystal structure, particle changes, and dissolution would be compared before and after irradiation, while sample temperature is monitored. Unirradiated samples, liquid-only samples, and heating controls would help distinguish changes caused by irradiation from those associated with the liquid or temperature.

These scans could be performed with a tunable mid-infrared free-electron laser. The FELBE sources at ELBE in Germany cover the required infrared range (Helm et al., 2023). The CTFEL upgrade in China is designed to extend its spectral coverage to 0.1–125 THz (Du et al., 2024). We plan to conduct irradiation experiments at CTFEL once the upgrade and commissioning provide access to the required frequencies.

If the initial tests yield reproducible crystal changes with controlled sample heating, subsequent biological tests would examine the effects of the treated material. Experiments in isolated tissue would then examine how light reaches the crystals through the fiber, how much tissue temperature rises, and whether nearby tissue is damaged. These observations would help select the fiber position and irradiation conditions for later preclinical *in vivo* studies.

## 5 Conclusions

Considering the limitations of pharmacological treatments for gouty arthritis, local and targeted physical therapies may complement established urate-lowering treatment. This study proposes a laser-targeted approach relying on PPRA. Based on the selection rules for photon–phonon coupling, a strong absorption region was identified for frequency-screening experiments. Simulations and analyses suggest scanning 1627–1705 cm^-^¹ with a tunable mid-infrared laser, using an initial center near 50 THz (~1666 cm^-^¹, photon energy 206.56 meV). The IR-active modes of the anhydrous model in this range also support testing continued absorption after structural water loss.

This study is primarily exploratory and limited to computational simulations. The potential for thermal damage to the skin and surrounding tissues makes temperature monitoring important in subsequent irradiation experiments. Measurements of absorption by the crystals and the surrounding medium, crystal changes, and tissue temperature could help establish suitable laser settings and fiber positions. Comparing the candidate frequencies with nearby off-resonance frequencies would show whether frequency selection reduces the energy needed for similar crystal changes.

The first step toward experimental validation of the proposed mechanism involves *in vitro* studies exposing synthetic MSUM crystals to tunable mid-infrared lasers to observe crystal changes under controlled conditions. These experiments would test the proposed photon–phonon resonant absorption mechanism, laying the groundwork for future biomedical collaborations.

## Data Availability

The raw data supporting the conclusions of this article will be made available by the authors, without undue reservation.
